# Rank Ordered Design Attributes for Health Care Dashboards Including Artificial Intelligence: Usability Study

**DOI:** 10.2196/58277

**Published:** 2024-11-20

**Authors:** Melina Malkani, Eesha Madan, Dillon Malkani, Arav Madan, Neel Singh, Tara Bamji, Harman Sabharwal

**Affiliations:** 1 Bullis School Potomac, MD United States; 2 George Washington University Washington DC, DC United States; 3 University of Pennsylvania Philadelphia, MD United States; 4 Basis Independent McLean Vienna, VA United States; 5 Duke University Durham, NC United States; 6 Scarsdale High School Scarsdale, NY United States; 7 St Stephens and St Agnes School Alexandria, VA United States

**Keywords:** data visualization, dashboards, public health, population health, informed decisions, consumer decision-making, health data, usability

## Abstract

**Background:**

On average, people in the United States visit a doctor 4 times a year, and many of them have chronic illnesses. Because of the increased use of technology, people frequently rely on the internet to access health information and statistics. People use health care information to make better-educated decisions for themselves and others. Health care dashboards should provide pertinent and easily understood data, such as information on timely cancer screenings, so the public can make better-informed decisions. In order to enhance health outcomes, effective dashboards should provide precise data in an accessible and easily digestible manner.

**Objective:**

This study identifies the top 15 attributes of a health care dashboard. The objective of this research is to enhance health care dashboards to benefit the public by making better health care information available for more informed decisions by the public and to improve population-level health care outcomes.

**Methods:**

The authors conducted a survey of health care dashboards with 218 individuals identifying the best practices to consider when creating a public health care dashboard. The data collection was conducted from June 2023 to August 2023. The analyses performed were descriptive statistics, frequencies, and a comparison to a prior study.

**Results:**

From May 2023 to June 2023, we collected 3259 responses in multiple different states around the United States from 218 people aged 18 years or older. The features ranking in descending order of importance are as follows: (1) easy navigation, (2) historical data, (3) simplicity of design, (4) high usability, (5) use of clear descriptions, (6) consistency of data, (7) use of diverse chart types, (8) compliance with the Americans with Disabilities Act, (9) incorporated user feedback, (10) mobile compatibility, (11) comparison data with other entities, (12) storytelling, (13) predictive analytics with artificial intelligence, (14) adjustable thresholds, and (15) charts with tabulated data.

**Conclusions:**

Future studies can extend the research to other types of dashboards such as bioinformatics, financial, and managerial dashboards as well as confirm these top 15 best practices for medical dashboards with further evidentiary support. The medical informatics community may benefit from standardization to improve efficiency and effectiveness as dashboards can communicate vital information to patients worldwide on critically prominent issues. Furthermore, health care professionals should use these best practices to help increase population health care outcomes by informing health care consumers to make better decisions with better data.

## Introduction

### Overview

A health care dashboard is a visual representation of vital health care data designed to emphasize key information for individuals and organizations, aiding them in making informed decisions [1]. These dashboards are increasingly used worldwide to track and report emerging and widespread diseases, trends, and other information to allow the public to make better health care decisions. [2,3]. Some examples include the Johns Hopkins COVID-19 dashboard, the Centers for Disease Control and Prevention FluView Interactive dashboard, the World Health Organization mpox dashboard, and the State of Pennsylvania’s Cancer Statistics dashboard.

Dashboards can effectively communicate health information to allow the public to make better health care decisions for themselves [4-6]. On the other hand, if health care dashboards are presented poorly or without the proper information, they can be ineffective tools for the public [7].

The research team conducted an observational review and surveys on the effectiveness of health care dashboards in the United States to better understand and improve their design elements. As a part of this research, the team reviewed over 250 US city, county, and state health care dashboards as an observational assessment of whether their designs were favorable or not. Along with the observational study, the team conducted 2 surveys with the public about the favorability of the top 15 best practices for both COVID-19 and health care dashboards. The COVID-19 dashboard survey had 118 people participating and the health care dashboard survey had 218 participants. The latter is the focus of this research article.

From these surveys and subsequent analysis, the authors were able to develop and confirm the top 15 best practices of health care dashboard design. These 15 top best practices were assessed as the most important aspects of a health care dashboard’s effectiveness.

There is a wide range of qualities when it comes to health care dashboard design. Some dashboards are confusing and difficult to read while others are clear and easily comprehensible. This study attempts to quantify what makes an effective health care dashboard.

With these best practices, practitioners can build upon these key elements to design and disseminate effective health care dashboards allowing the public to make better health care decisions and in turn, help improve health care outcomes.

The aim of this study is to rank and explain the relative importance of aspects of health care dashboards so that they can be more easily usable and understandable.

### Background

The average person in the United States visits a doctor 4 times a year, and 6 in 10 adults in the United States have a chronic disease [8]. In total, 61% of all US adults have searched for health or medical information on the internet [9]. In addition, 49% have accessed a website that provides information about a specific medical condition or problem [3]. Therefore, it is important that health care information used by the public is both informative and easily understood, since many people are constantly searching for new health care information.

The public makes health care decisions based on available information, either sourced from the internet or received in-person. Public health tools such as health care dashboards play a key role in the health care decision-making process because they can be readily available and provide real-time information instantly. Doctors and patients can benefit from health care dashboards, if they are designed in the best interest of the public and health care officials.

An effective health care dashboard should effectively display relevant and comprehensible information to its users [10,11]. For instance, a health care dashboard on cancer that provides data promoting timely screenings for breast and colon cancer can not only contribute to reducing the prevalence of these cancers, but also may influence patients in the consideration of appropriate therapy, enabling them to make well-informed decisions in a collaborative manner. It is also important that information and data are offered through assorted designs to help all demographics, including minorities and ethnically diverse populations that are more susceptible or at risk of specific public health issues [1]. For example, Hispanic individuals are about 50% more likely to die from diabetes or liver disease [12]. Dashboards that prioritize population-specific data for screening and prevention in the Spanish language may enhance usability leading to improved prevention strategies.

Furthermore, broadening the conversation regarding the application of these design features in various health care settings, such as managing chronic diseases, tracking epidemics, and incorporating artificial intelligence (AI) and predictive analytics, could further enhance the practical implications and overall impact of this research in the health informatics field.

### Role of AI and Predictive Analytics

Furthermore, the use of AI to display dynamic on-demand predictive analytics in a health care dashboard can have a significant potential future impact. Its incorporation in dashboard design is especially helpful for analyzing and displaying data, identifying trends, forecasting future health scenarios, and providing personalized recommendations based on historical data and current health indicators.

The integration of AI significantly impacts usability by offering enhanced decision support, improving efficiency, personalizing user experiences, increasing accessibility, and enabling data-driven health interventions. These factors make health care dashboards more powerful tools for helping individuals and organizations make informed health decisions, leading to better health care outcomes. By addressing the effective integration and potential effects of AI, this research offers important perspectives on the future of dashboard advancement in the health care industry.

The study aims to provide a reliable assessment of the key attributes that make health care dashboards effective tools for public health decision-making including the use of AI for predictive analytics and other prominent features.

## Methods

### Overview

We initially examined over 250 public-facing health care dashboards from both the US government and commercial sources such as the Johns Hopkins COVID-19 dashboard, the Centers for Disease Control and Prevention FluView Interactive dashboard, the World Health Organization Healthcare mpox dashboard, and the State of Pennsylvania’s Cancer Statistics dashboard. After the review of these health care dashboards, the authors identified key design elements for health care dashboards and then conducted a survey validating the top 15 attributes to produce a rank-ordered list.

The attributes surveyed are as follows: (1) easy navigation, (2) high usability, (3) use of diverse chart types, (4) mobile compatibility, (5) predictive analytics with AI, (6) incorporated historical data, (7) use of clear descriptions, (8) compliance with the Americans with Disabilities Act (ADA), (9) comparison data with other entities, (10) adjustable thresholds, (11) simplicity of design, (12) consistency of data, (13) incorporated user feedback, (14) storytelling, and (15) charts with tabulated data.

In a previous study, we gathered responses from 118 individuals for 10 key elements on COVID-19 dashboards [2]. For this new and expanded survey, we surveyed 218 participants and introduced 5 additional attributes not previously explored in our research. These newer attributes are storytelling, predictive analytics leveraging AI, mobile compatibility, incorporated historical data, and consistency of data.

This survey was distributed using a combination of web-based platforms and direct outreach to ensure a wide and diverse demographic representation. The anonymous Google Forms survey was administered to the public by either providing a tablet device to employees and patients in medical clinics in Germantown, MD, Rockville, MD, Charlestown, WV, and Lansdowne, VA as well as an email sent to participants nationwide. The online survey stated the benefits of the healthcare dashboard study and informed the participants that the results collected were anonymous. The target population was adults over the age of 18 in the United States.

### Data Analysis

This survey was distributed using a combination of web-based platforms and direct outreach to ensure a wide and diverse demographic representation. Participants who did not respond were sent a follow-up email. Measures were taken to minimize response biases by ensuring anonymity, randomizing the order of questions, and providing clear and neutral question wording. In addition, the survey was pretested with a small group to identify and rectify any potential sources of bias or confusion. The survey consisted of 15 questions. Responses of “yes” counted as 1 point, while responses of “no” counted as 0 points. The data from the survey was imported into Excel and online calculators by two different operators to perform the statistical analysis. These analyses were frequencies, descriptive statistics, and a comparison of design attributes with a prior study.

### Demographic Information

Demographic data including county, state, and gender were included for all survey participants. Survey respondents represented a diverse range of 10 US states, including Maryland, Virginia, and Texas. The team aimed to ensure that they included at least 5 different US states. In accordance, the selection process satisfied the team’s candidate attributes, as data were collected from a larger number of states than originally contemplated.

### Ethical Considerations

Online data collected from this study were anonymous. A waiver from the Bullis School ethics committee was granted retrospectively.

## Results

### Overview

The survey aimed to help the impact of web-based learning through data dashboards on all health care information. Previous studies have highlighted how to easily read dashboards instead of the specific dashboard designs that would benefit users. The survey was developed using credible questionnaire resources, such as Google Forms and Survey Monkey, and tailored to our study needs. This survey was pretested with a small group of students to receive feedback and improve clarity and flow. The inclusion criteria were that all participants had to be aged 18 years or older and a total of 3259 responses were recorded (Table 1). The number of questionnaire respondents was 218 and the response rate was 89% (244/218). The authors conducted a survey of 218 individuals aged 18 years or older. The authors calculated a total of 3259 responses with 2945 responses of “yes” and 314 responses of “no.”

The “use of charts with tabulated data” had the lowest percent agreement of “yes” responses of 83% (182/218), whereas “easy navigation” had the highest percent agreement of “yes” responses of 96% (209/218), and the “use of predictive analytics using AI” had “yes” responses of 87% (188/218), ranking at the 13th most popular attribute (Table 2).

Textbox 1 shows the five new dashboard attributes added to this study in comparison to the prior Malkani study on dashboards. These new attributes incorporate new technologies, such as mobile and artificial intelligence and 3 other attributes comprising the top 15 attributes design attributes for health care dashboards. The following sections provide a discussion for each of the 15 design attributes.

**Table 1:** Demographic results of the public health dashboard design survey, 2023 (N=218).

Measure and item		Individuals, n (%)
**Sex**		
	Male	82 (61.9)
	Female	135 (37.6)
**Gender**		
	Nonbinary	1 (0.5)
**Age (years)**		
	18-29	61 (28.1)
	30-39	25 (11.5)
	40-49	54 (24.9)
	50-59	26 (12)
	60-69	18 (8.3)
	70-79	26 (12)
	80-89	6 (2.8)
	90+	1 (0.5)
**State of residence**		
	Maryland	46 (21.2)
	West Virginia	70 (32.4)
	Virginia	45 (20.8)
	New Jersey	22 (10.1)
	Pennsylvania	12 (0.056)
	Missouri	8 (0.037)
	Iowa	3 (0.014)
	Texas	2 (0.009)
	Ohio	2 (0.009)
	South Carolina	3 (0.014)
	Indiana	1 (0.005)
	Florida	1 (0.005)
	Tennessee	1 (0.005)

**Table 2:** Results of health dashboard design survey, 2023 (N=218).

Rank	Dashboard feature	“No” (0; mean 21, SD 8, range 9-36; median 21, IQR 15-25)	“Yes” (1; mean 197, SD 8, range 182-209; median 195, IQR 193-202)	Total points (mean 217, SD 1, range 215-218; median 217, IQR 217-218)	Percentage of agreement (%; mean 91%, SD 4%, range 83%-96%; median 91%, IQR 88%-94%)
1	Easy navigation	9	209	218	96
2	Incorporated historical data	10	207	217	95
3	Simplicity of design	14	204	218	94
4	High usability	14	204	218	94
5	Use of clear descriptions	16	201	217	93
6	Consistency of data	16	201	217	93
7	Use of diverse chart types	23	195	218	91
8	Compliance with the Americans with Disabilities Act	20	195	215	91
9	Incorporated user feedback	21	195	216	91
10	Mobile compatibility	23	194	217	89
11	Comparison data with other entities	26	192	218	88
12	Storytelling	24	193	217	88
13	Predictive analytics with artificial intelligence	29	188	217	87
14	Adjustable thresholds	33	185	218	85
15	Charts with tabulated data	36	182	218	83
Total		314	2945	3259	90

**Key design attributes for public facing health care dashboards including 5 new attributes as bolded in a usability study, 2023 (N=218).**

**The top 15 medical dashboard design attributes (N=218)**

- Easy navigation
- Incorporated historical data
- Simplicity of design
- High usability
- Use of clear descriptions
- Consistency of data
- Use of diverse chart types
- Compliance with the Americans with Disabilities Act
- Incorporated user feedback
- Mobile compatibility
- Comparison data with other entities
- Storytelling
- Predictive analytics with artificial intelligence
- Adjustable thresholds
- Charts with tabulated data

### Easy Navigation

The ability to navigate through various pages and windows of a health care dashboard with ease is an essential and top-rated element of a health care dashboard. Users of health care dashboards should be able to navigate fully throughout the dashboard regardless of education level or background [13,14]. While reviewing over 250 different health care dashboards, it was noted that many of the dashboards were difficult to navigate due to erroneous navigation features such as unclickable links and no scroll bars. We noted that several modifications could be introduced in some of the dashboards regarding the dimensions and positioning of the navigation elements, aligning them more closely with the more efficient dashboards or data services like those found in Apple or Samsung mobile phones. In addition, a health care dashboard should allow a user to hover over a data element to review additional information regarding that element of data, known as a “focus mode” [15] for a data dashboard or website. Easy navigation can be a major key to the success of health care dashboards and should be advanced further in many health care dashboards. It ranked as the #1 design attribute out of 15 design attributes based on the 218-person survey.

### High Usability

High usability for health care dashboards is a key concept for dashboard design. The attribute of high usability can be achieved through faster loading speeds, consistent labeling, simpler layouts, and optimization for multiple devices. Poor responsiveness of the dashboard can negatively affect the perception of the quality and reliability of your dashboard. Using simple, clear, and consistently labeled data with highly readable fonts and text, effective labeling, and avoiding clutter and distortion helps optimize usability [16-18].

A good dashboard should have a simple layout with a logical and coherent structure, with a clear hierarchy, flow, and balance. Using grids, white space, borders, and headings to create visual separation and alignment among elements can help create harmony and proportion on the dashboard, helping the user process the information quickly. Finally, health care dashboards with high usability should have accurate, clear, and concise information to increase the adoption and use of the dashboard and its information.

### Use of Adjustable Thresholds

An adjustable threshold customizes the level of specificity that a graph or other charts show and makes the dashboard more interactive. The use of interactive and adjustable thresholds is a principal factor to incorporate in health care dashboards. They are advantageous as users can interact with the dashboard and modify its parameters through these adjustable thresholds. For example, a short 10-day period may not be able to capture noticeable fluctuations in COVID-19 or mpox cases [15,19]. Expanding the view, a 90-day threshold, for example, brings benefits when assessing the dashboard more comprehensively. Incorporating adjustable thresholds based on percentages offers greater flexibility enabling easier and effective evaluation of the data. Transitioning from daily to monthly statistics or changing data trends over an extended period helps improve insights. This method proves significantly more potent, helping streamline data evaluation.

The interactivity of the dashboards makes the experience more intuitive, responsive, and meaningful, allowing the user to filter, drill down, zoom, highlight, and compare data as needed.

### Use of Diverse Types of Charts

In a health care dashboard, incorporating a diverse set of chart types offers many advantages. To diversify visualization options and expand the scope of the information presented, it is important to offer a variety of chart types such as bar, line, and pie charts. Considering the diverse range of dashboard users, the addition of elements other than line charts, such as heat maps, can add variety, and help with comprehension by different user groups. Studies show that users prefer to review various chart types. In addition, interactive graphs that allow users to explore data from different perspectives can be valuable additions.

### Use of Charts With Tabulated Data

The use of charts with tabulated data can be immensely helpful because it improves data comprehension, enables the ability to see and track trends in the data, and promotes data exploration. Tables allow a deeper dive into the numbers and help examine exact values instead of focusing on approximations or visualizations. For example, displaying the number of illnesses diagnosed over a specific period may be easier to accomplish with a chart by simply showing numbers and locations. However, a tabulated format is more useful when it is necessary to communicate other additional variables as well such as causes, outcomes, and even specifics about the length of the illness, number of relapses, and more.

### Incorporated User Feedback

Health care dashboards are powerful tools, but they are not static. As a best practice, they should be constantly updated and improved based on feedback from the user [13]. User satisfaction and feedback are important concepts for the design of health care dashboards. While the designers of dashboards try to anticipate the concerns of users, it is vital to have a quick and simple system for users to express their suggestions about the dashboard. With a similar system, designers can incorporate feedback into their dashboards and attract more user activity. By applying user feedback, designers of health care dashboards can optimize the comprehensibility of their information. User feedback provides designers with valuable insights into user perspectives, helping them identify potential enhancements, and rendering data more accessible and comprehensive. Designs that incorporate feedback from the user and continuously monitor and evaluate the feedback data help keep the dashboard updated, relevant, and user-friendly.

### Simplicity of Design

Simplicity of design is a key factor for creating a well-designed health care dashboard. Users should be able to navigate any health care dashboard easily and effectively. Based on the authors’ survey of 218 adults, the design of health care dashboards should not be complex. The amount of text on each page should be kept to a minimum and information should be presented simply and concisely. The most important and urgent information should be shown on the first pages while less relevant information should be displayed later. Users should be able to search for more details as they navigate the dashboard. The simplicity of design allows all users, regardless of their age or background, to easily interpret and analyze data.

### Adding Clear Descriptions for Charts

Since data displayed in charts can be overwhelming, complicated, or difficult to understand, it is important that there are clear and concise descriptions for charts. Descriptions should be simple, while still getting the information across. With clear axis labels, units, and scales of measure, as well as titles, the users can easily comprehend the data.

### Compliance With the ADA

To ensure equal access to information to all people across the United States, health care dashboards should make efforts to abide by the ADA. For example, there are an estimated 300 million people in the world with color vision deficiency and red-green color blindness is the most common form of color blindness [20]. To address this issue, health care dashboards should refrain from using red and green colors. The dashboards should make sure that the alerting system is also differentiated in some other way besides color, for example, pairing light and dark colors or light and dark variations of individual colors. Health care professionals’ dashboards should use the ADA website to ensure equal access to health care dashboards for all people across the United States.

### Comparison of Data With Other Entities

Comparison of data with other reference groups is essential for a health care dashboard to be easily read and understood. By comparing and displaying different elements such as demographics users can compare their own experiences with other groups in similar situations, proving a valuable tool toward better comprehension. For example, the number of mpox cases in Canada versus the United States displayed on a dashboard allows users to understand the prevalence and potential risk of mpox in the context of their own lives and locality. It can also warn or help make educated predictions about what could potentially arise in the future due to the number of cases or deaths in a neighboring area. Dashboards are intended to inform the public about information that should help them make informed decisions not only about the current situation but also the future.

### Historical Data

Comparison with historical data on a health care dashboard is highly effective because it allows for meaningful comparisons in terms of any changes. Providing historical data on health care dashboards will enable users to identify and analyze specific trends and patterns to make their own decisions based on past experiences. Being able to track data throughout the years and using it to make future decisions makes historical data important in a variety of health care–related situations.

### Storytelling

When presenting data, storytelling is vital for building relevance and aiding in comprehension. When a person reviews a chart or selection of data in a limited and isolated manner it can lead to a lack of comprehension. Converting the data within the context of the background helps create a narrative helping in relevance and comprehension. For example, a story can be presented through points on the graph, explaining a particular event and why it affected the data, or through creating a section outside of the data to explain all factors that could have created trends that were shown. Through storytelling, a viewer can fully grasp and better understand the information. It also helps create a “timeline” of events, as well as a link to future events or actions that may affect the data in question.

### Consistency of Data

The consistency of data in health care dashboards is crucial for accurate and reliable information. Consistency refers to the quality of the data and in reference to data being accurate, uniform, dependable, and up-to-date throughout. Maintaining consistency ensures that the data presented on the dashboard are trustworthy and can be used for informed decisions. Throughout health care dashboards, data are collected from complicated systems and sources, and consistency becomes one of the most crucial factors to help in comprehension. As an example, consistency should be shown through the different axes of the charts having the same scale of measure. Consistency of data enables health care professionals, policymakers, and researchers to analyze trends and make informed decisions based on accurate, comparable, and reasonable data. Most importantly the consistency of data in health care dashboards affirms the credibility of the information provided and improves the effectiveness of the data.

### Predictive Analytics With AI

As our world continues to develop, predictive analytics based on AI technology is increasingly relevant and beneficial. It is only fitting that predictive analytics using AI should be applied to the field of health care informatics research. If the user can input certain individual parameters, the dashboards should be able to provide predictive analytics for any parameter or question. Using predictive analytics, analysts can examine how data trends may fare in the future, and a user can review a detailed example of future data [21]. Predictive analytics of potential future trends are important for dashboards and data as they allow users to make decisions for themselves beyond the expectation of a linear trend. When viewing data for even a few past years, consumers assume linear trends and make predictions based on their perception of what data will look like in the future. Performing a similar analysis with predictive analytics with AI will provide more accurate predictions and allow people to make better medical decisions.

### Mobile Compatibility

The best health care dashboards are designed with mobile compatibility. Health informaticians increasingly aim to make health care dashboards accessible and usable on diverse types of devices. Mobile compatibility is important given that most users spend a great deal of time viewing and accessing information on their mobile devices. Mobile compatibility is important for 3 reasons. First, mobile compatibility empowers users with the ability to access critical data anytime and anywhere allowing real-time data tracking, timely updates, and retrieval. Second, mobile compatibility ensures that charts and graphs seamlessly adjust to the screen dimensions of the user’s device. This adaptation optimizes data visualization and user experience by presenting data in an easily comprehensible format, regardless of the screen size. Finally, mobile compatibility enables health care dashboards to leverage the unique functionalities of different devices. For instance, when data are accessed on a smartphone, these dashboards can use features like “push notifications” to provide users with instant updates and keep them informed about the latest health care information. Given the widespread use of mobile devices in society, it is imperative to publish health care dashboards compatible with a range of devices, from large public displays to compact cell phones, thereby promoting broader accessibility to health care data.

## Discussion

### Principal Findings

Health dashboards are an important part of the health-data world which provides insight into health information through easily accessible images, charts, and representations. We found that the top 5 most important design attributes for an effective health care dashboard are easy navigation, simplicity of design, high usability, use of clear descriptions, and use of diverse chart types. The results from this research produce the top 15 design attributes for an effective health care dashboard. As health care dashboards are better designed, the idea is that the public will make better-informed health care decisions which will in turn increase health care outcomes. These specific 15 design attributes have been generally discussed in previous literature for health care dashboards, but this is the first time they have been specifically identified for health care dashboards. Similar studies have supported this overall research stream but do not compete with or overlap with these results. AI-generated dashboards in particular may have certain ethical biases that would lead to potentially incorrect conclusions by the public. The user of the dashboard should be informed if AI has been used in predictive analytics, for example, and the characteristics of the data (ie, size, type, kind, and location) should be available for the user to access to address the ethical concerns about the use of AI in health care.

The findings add to the prior literature on the design attributes for healthcare dashboards [2, 10, 15, 19, 21, 22]. As prior publications emphasize the importance of healthcare dashboards [4, 6, 17], this study focused on the design characteristics of healthcare dashboards.

Ansari et al [21] stated that usability problems exist with public health dashboards. Their checklist can be used in concert with these best practices for the healthcare dashboards. Karami at al [10] developed 7 key concepts and criteria for effective dashboards, whereas this study provides 15 best practices for healthcare dashboards. Murphy et al [13] stated that informatics and human factors principles should be incorporated into dashboard design. We provide human factor and informatics factors in the best practices for the design of dashboards. Malkani et al [2] developed best practices for the design of COVID dashboards. This study builds on those results and is focused on the broader topic of healthcare dashboards. Khodaveisi at al [19] provided the characteristics of COVID dashboards like Malkani. Finally, Martins et al [15] provide the best practices for dashboards for business management. These studies are consistent with this study and the prior literature is not in conflict with the results.

### Limitations

Over 70% of the study’s participants (161/218) were from the 3 US states including Maryland, West Virginia, and Virginia. The study, therefore, is limited as far as the generalizability across the entire United States is concerned. Furthermore, with only 218 participants the study could have had a greater impact if it were for example over 1000 participants with at least 5 to 10 participants per state. Beyond the number and location of the participants, this study is limited in scope as it is based on self-reporting by the public for what they consider a positive dashboard design. It does not measure whether a better dashboard design, for example, results in a better actual health care decision. Beyond these limitations, this study is important as it attempts to quantify the top 15 elements of health care dashboard design which is undocumented in this context in previous literature.

### Conclusion

As technology evolves, the availability of resources and data has become increasingly easier and better. With a click or a quick search, consumers have access to an abundance of health care data and data dashboards which aid in making informed health care decisions. However, health care dashboards may not be of the highest quality or as easily understood. Through our observational review and multiple surveys, we evaluated the effectiveness of health care dashboards in the United States to better understand and improve their design elements. From our analysis, we were able to develop and confirm the top 15 best practices of health care dashboard design from the ease of navigation to the use of predictive analytics. These 15 top best practices were assessed as the most important aspects of a health care dashboard’s effectiveness. The studies validated and concluded that the top 5 attributes of health care dashboards, such as easy navigation, simplicity of design, high usability, use of clear descriptions, and use of diverse chart types. As identified and analyzed, best practices can be incorporated in order to design and disseminate effective health care dashboards making valuable health care information available to the public. The availability of better health care dashboards will help consumers make better and more informed health care decisions resulting in better health care outcomes.

## Abbreviations

ADA: Americans with Disabilities Act
AI: artificial intelligence

## References

[1] Dixon BE, Dearth S, Duszynski TJ, Grannis SJ (2022). Dashboards are trendy, visible components of data management in public health: sustaining their use after the pandemic requires a broader view. Am J Public Health.

[2] Malkani D, Malkani M, Singh N, Madan E (2023). Best practices for the design of COVID-19 dashboards. Perspect Health Inf Manag.

[3] Alanazi A (2023). Clinicians' views on using artificial intelligence in healthcare: opportunities, challenges, and beyond. Cureus.

[4] Bakken S (2022). Meeting the information and communication needs of health disparate populations. J Am Med Inform Assoc.

[5] Gleeson J, Kitchin R, McCarthy E (2022). Dashboards and public health: the development, impacts, and lessons from the Irish government COVID-19 dashboards. Am J Public Health.

[6] Katapally TR, Ibrahim ST (2023). Digital health dashboards for decision-making to enable rapid responses during public health crises: replicable and scalable methodology. JMIR Res Protoc.

[7] Helminski D, Kurlander JE, Renji AD, Sussman JB, Pfeiffer PN, Conte ML, Gadabu OJ, Kokaly AN, Goldberg R, Ranusch A, Damschroder LJ, Landis-Lewis Z (2022). Dashboards in health care settings: protocol for a scoping review. JMIR Res Protoc.

[8] (2023). COVID-19 vaccines for children and teens. CDC.

[9] Wang X, Cohen RA (2023). Health information technology use among adults: United States, July–December 2022. NCHS Data Brief.

[10] Karami M, Langarizadeh M, Fatehi M (2017). Evaluation of effective dashboards: key concepts and criteria. Open Med Inform J.

[11] Ullrich A, Eckelmann F, Ghozzi S (2019). Dashboards as strategy to integrate multiple data streams for real time surveillance. Online J Public Health Inform.

[12] Martell BN, Garrett BE, Caraballo RS (2016). Disparities in adult cigarette smoking - United States, 2002-2005 and 2010-2013. CDC.

[13] Zhuang M, Concannon D, Manley Ed (2022). A framework for evaluating dashboards in healthcare. IEEE Trans Vis Comput Graph.

[14] Buttigieg SC, Pace A, Rathert C (2017). Hospital performance dashboards: a literature review. J Health Organ Manag.

[15] Martins N, Martins S, Brandão D, Raposo D, Neves J, Silva J (2021). Design principles in the development of dashboards for business management. Perspectives on Design II.

[16] Elshehaly M, Randell R, Brehmer M, McVey L, Alvarado N, Gale CP, Ruddle RA (2021). Qualdash: adaptable generation of visualisation dashboards for healthcare quality improvement. IEEE Trans Vis Comput Graph.

[17] Gallifant J, Kistler EA, Nakayama LF, Zera C, Kripalani S, Ntatin A, Fernandez L, Bates D, Dankwa-Mullan I, Celi LA (2023). Disparity dashboards: an evaluation of the literature and framework for health equity improvement. Lancet Digit Health.

[18] (2021). New Estimates of People Diagnosed / Treated for Lyme: 476,000 Annually.

[19] Khodaveisi T, Dehdarirad H, Bouraghi H, Mohammadpour A, Sajadi F, Hosseiniravandi M (2023). Characteristics and specifications of dashboards developed for the COVID-19 pandemic: a scoping review. Z Gesundh Wiss.

[20] About Colour Blindness.

[21] Ansari B, Martin EG (2022). Development of a usability checklist for public health dashboards to identify violations of usability principles. J Am Med Inform Assoc.

[22] Murphy DR, Savoy A, Satterly T, Sittig D, Singh H (2021). Dashboards for visual display of patient safety data: a systematic review. BMJ Health Care Inform.

